# Facile Approach to Develop Hierarchical Roughness fiber@SiO_2_ Blocks for Superhydrophobic Paper

**DOI:** 10.3390/ma12091393

**Published:** 2019-04-29

**Authors:** Qing Wang, Jieyi Xiong, Guangxue Chen, Ouyang Xinping, Zhaohui Yu, Qifeng Chen, Mingguang Yu

**Affiliations:** 1 YUTO Research Institute, Shenzhen YUTO Packaging Technology Co., Ltd., Shenzhen 518000, China; wangqing@szyuto.com (Q.W.); alex.yu@szyuto.com (Z.Y.); 2 State Key Laboratory of Pulp and Paper Engineering, South China University of Technology, Guangzhou 510640, China; xjy18211323790@163.com (J.X.); qfchen@scut.edu.cn (Q.C.); 3 School of Chemical and Energy Engineering, South China University of Technology, Guangzhou 510640, China; ceouyang@scut.edu.cn; 4 School of Materials Science and Energy Engineering, Foshan University, Foshan 528000, China; mesyumg@fosu.edu.cn

**Keywords:** cellulose fibre, superhydrophobic paper, self-cleaning, composite materials

## Abstract

Papers with nanoscaled surface roughness and hydrophobically modification have been widely used in daily life. However, the relatively complex preparation process, high costs and harmful compounds have largely limited their applications. This research aims to fabricate superhydrophobic papers with low cost and nontoxic materials. The surface of cellulose fibers was initially coated with a film of SiO_2_ nanoparticles via sol-gel process. After papermaking and subsequent modification with hexadecyltrimethoxysilane through a simple solution-immersion process, the paper showed excellent superhydrophobic properties, with water contact angles (WCA) larger than 150°. Moreover, the prepared paper also showed superior mechanical durability against 10 times of deformation. The whole preparation process was carried out in a mild environment, with no intricate instruments or toxic chemicals, which has the potential of large-scale industrial production and application.

## 1. Introduction

Cellulose based papers have been widely used in product packaging for their advantages of being renewable, recyclable and biodegradable compared with petroleum-based materials. However, the high moisture content and moisture absorption of cellulose fiber lead to poor physical property of paper-based materials [1]. So, it is of vital importance to endow cellulose based paper with the hydrophobic property. Tradition methods for the fabrication of hydrophobic paper were mainly based on surface coating with waxy derived materials [2,3,4,5] such as alkyl ketene dimer, carnauba wax, and beeswax. The thermal instability of the wax largely limited the application of the papers.

With the development of interfacial chemistry, materials with superwetting properties have attracted wide attention from both academia and industry due to their potential applications in antibacterial property [6,7], self-cleaning [8,9,10], printing and reprography [11,12,13,14], separation of liquids [15,16,17], etc. It is well known that certain surface roughness and low surface energy material modification are two key elements for constructing superhydrophobic interface [18,19,20,21,22,23,24,25]. Methods have been developed to build superhydrophobic papers [26,27,28,29,30,31,32,33,34,35], which could be basically categorized as chemical deposition, colloidal assemblies, layer by layer deposition, etc. The complicated procedure, high cost and harmful compounds have largely limited their applications. The construction of novel, high-efficiency, and economical superhydrophobic cellulose based paper, without destroying the pristine structure of the cellulose fiber, is still a great challenge.

**Figure 1:**
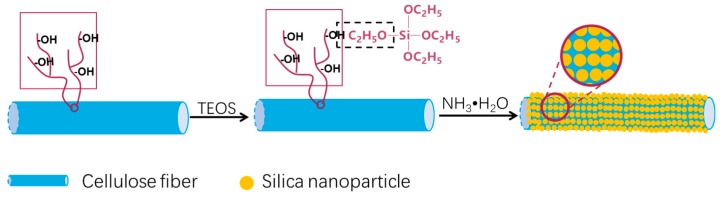
Schematic procedure of cellulose fiber-based superhydrophobic paper.

Hence, we present a facile method for converting cellulose fiber into superhydrophobic paper, with excellent superhydrophobic and self-cleaning properties, involving the use of biomass SiO_2_ modified cellulose fibers. As shown in Figure 1, cellulose fibers were used as raw material and treated with Tetraethoxysilane (TEOS) via the Stöber method aimed at constructing fibers@SiO_2_ hierarchical roughness surface structure. After the papermaking process and subsequent grafting with Hexadecyltrimethoxysilane (HDTMS), superhydrophobic paper was obtained. The chemical composition, surface morphology and wettability of the as-prepared paper were studied. Moreover, the self-cleaning and finger friction resistance performance were also evaluated in a simple way. This work could provide a facile and efficient approach to prepare superhydrophobic paper from low-cost and biodegradable cellulose fibers in nature.

## 2. Materials and Methods

### 2.1. Materials

Bleached hardwood pulp (75 SR°) was kindly provided by Chenhui Paper Co., Ltd (Guangzhou, China). Tetraethoxysilane (TEOS, >99%) and ammonium hydroxide (NH_3_•H_2_O, 25 wt.%) were purchased from Guangzhou Chemical Reagent Factory (Guangzhou, China). Hexadecyltrimethoxysilane (HDTMS, >99%) was purchased from Shanghai Macklin Biochemical Co., Ltd (Shanghai, China). Ethanol was provided by Sinopharm Chemical Reagent Co., Ltd. (Shanghai, China). Deionized water was homemade. All materials were used as received.

### 2.2. Synthesis of Silica Modified Cellulose Fibres

Silica modified cellulose fibers were prepared via a modified Stöber method [36]. In a typical procedure, 0.5 g of dry cellulose fibers (75 SR°) and 100 mL ethanol were added into a 250 mL three-neck round bottom flask with gentle stirrer until materials were completely dispersed. Then 1 mL of TEOS and a certain amount of ammonia were added to the above solution and reaction for at least 6 h at 30 °C. After being separated by centrifugation and washed with EtOH and water 3 times, the silica modified cellulose fibers composites were prepared.

### 2.3. Fabrication of Superhydrophobic Paper

Cellulose fibers@SiO_2_ based paper with 60g/m^2^ was first prepared via suction filtration. Then the as-prepared paper was added to a solution containing 0.5 mL of HDTMS and 45 g of EtOH and maintained for about 10 min, followed by drying under a vacuum at 50 °C for 12 h.

### 2.4. Characterization

FT-IR spectra were recorded on a VERTEX 70 IR spectrophotometer (Bruker Instruments, Karlsruhe, Germany). X-ray photoelectron spectroscopy (XPS) was analyzed using an Axis Ultra DLD multifunction X-ray photoelectron spectrometer (Kratos Instruments, Manchester, UK) with Al Ka radiation (20 eV) as the exciting source. Thermo-gravimetric analysis (TGA) was performed with TGA Q500 TA instrument (TA Instruments, PA, USA). Samples were heated at a ramp of 20 °C/min in nitrogen, with temperature ranges from 40 °C to 600 °C. The morphology of the surfaces was carried out on a Thermal field-emission scanning electron microscope (FESEM, Quanta 400F, Hillsboro, OR, USA) at 15 kV. Static water contact angles (WCA) were measured on Dataphysics OCA40 Misco (Dataphysics, Filderstadt, Germany) with liquid droplets of 5 μL. All the contact angles were determined by averaging values measured at least 3 different points on each sample surface.

## 3. Results

### 3.1. Composition Analysis

**Figure 2:**
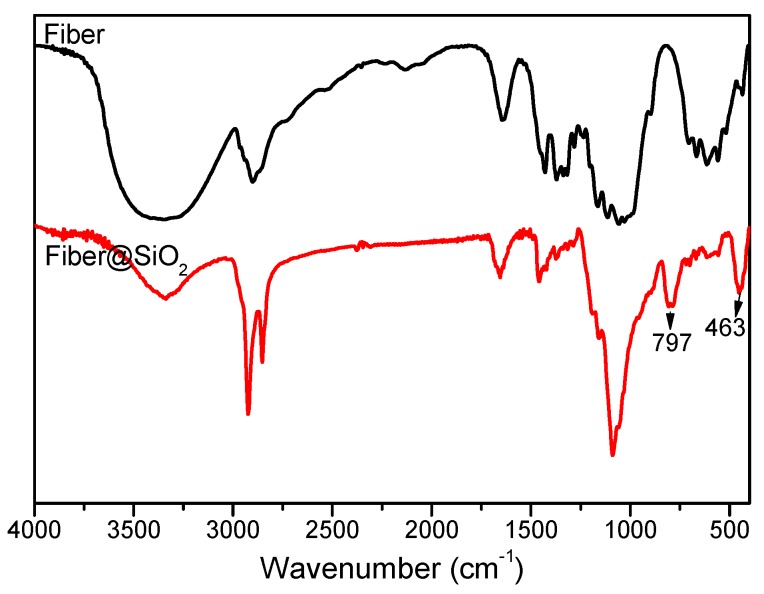
FT-IR spectra of raw cellulose fiber and SiO_2_ modified cellulose fiber.

FT-IR structures of raw cellulose fiber and fiber@SiO_2_ are shown in Figure 2. As for raw cellulose fiber, the vibration bands at 2910 cm^−1^ and 2855 cm^−1^ can be assigned to the asymmetrical and symmetrical stretching of –CH_2_, respectively. After in situ hydrolysis of TEOS, newly appeared symmetric etching vibration and bending vibration bands at 463cm^−1^ and 797cm^−1^ were assigned to Si–O characteristic peaks. In addition, a strong and wide absorption peak at 1084cm^−1^ attributed to Si–O-Si linkages, which indicated that silica was successfully carried on fibers [37]. 

**Figure 3:**
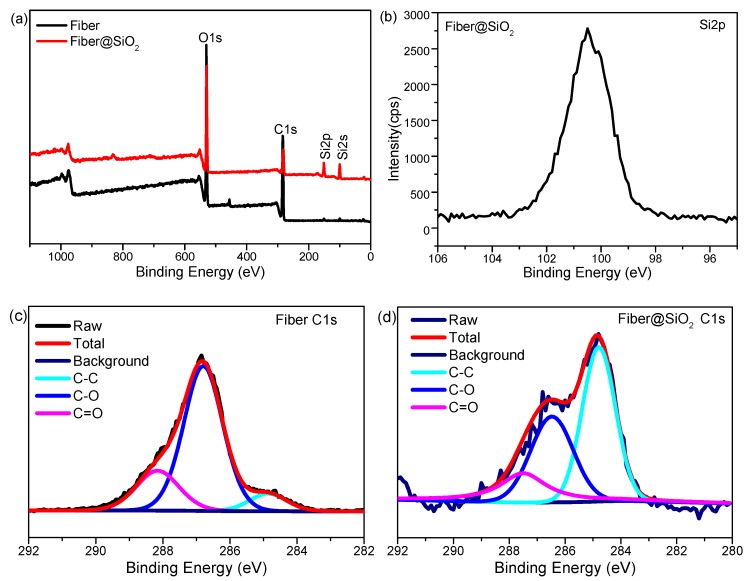
XPS spectra (**a**) and high resolution XPS spectra of Si 2p (**b**), C 1s (**c**,**d**) of raw cellulose fiber and SiO_2_ modified cellulose fiber.

XPS spectra was employed to determine the surface composition of SiO_2_-functionalized cellulose fibers, shown in Figure 3. XPS spectra of raw cellulose fibers showed C1s peak and O1s peak with binding energy at 284.8 eV and 532.4 eV, respectively. For the SiO_2_ modified cellulose fibers, new peaks appeared at 150 eV and 103 eV, which were attributed to Si2p and Si2s signals [38,39], respectively. Besides, the relative atomic concentration of carbon and oxygen decreased from 55.09% and 44.91% to 33.36% and 38.70% respectively, together with the appearance of Si 2p with 27.93%, which further confirmed the presence of SiO_2_ particles on the fiber surface (Table 1). Moreover, a high-resolution C 1s spectra of cellulose fibers before and after SiO_2_ modification were also performed (Figure 3c,d). The –C=O, C–O and C–C peaks of pristine fiber were located at 288.0, 286.5 and 284.8 eV, respectively [40]. After SiO_2_ modification (Figure 3d), the relative intensity of the peak for C–C increased due to the forming cellulose–(OH)Si(OC_2_H_5_)_3_ on cellulose fibers, which prove that silica was successfully grafted to the surface of the fiber, due to the hydroxy on cellulose.

**Figure 4:**
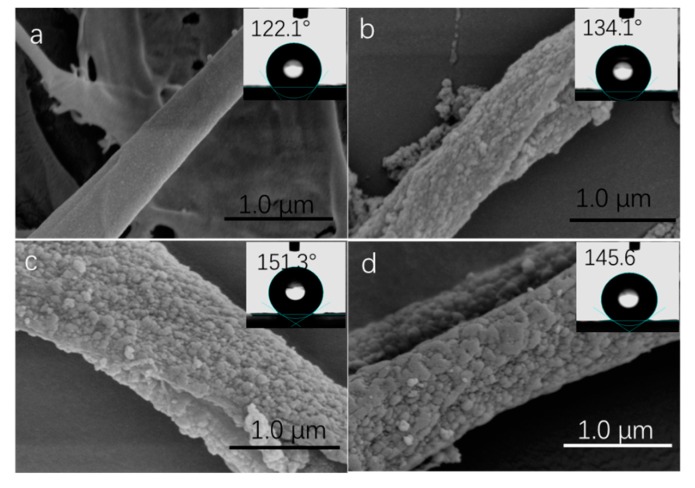
SEM images of raw cellulose fibers (**a**) and cellulose fibers catalyzed with different amount of ammonia: (**b**) 1 mL, (**c**) 2 mL, (**d**) 3 mL.

Morphologies of raw cellulose fibers and SiO_2_ modified cellulose fibers catalyzed with different amounts of NH_3_•H_2_O were examined by SEM shown in Figure 4. As shown in Figure 4a, the surface of raw cellulose fiber was smooth with natural grooves and veins. After in situ hydrolysis of TEOS, silica clusters appeared on the surface of the fiber, making the fiber surface rough and thus generating a dual-size surface structure. In addition, it is found that, as the NH_3_•H_2_O increased from 1 mL to 2 mL and 3 mL, the silica particles become bigger and more numerous, and even non-spherical particles were formed, possibly due to the aggregation of smaller particles [41].

**Figure 5:**
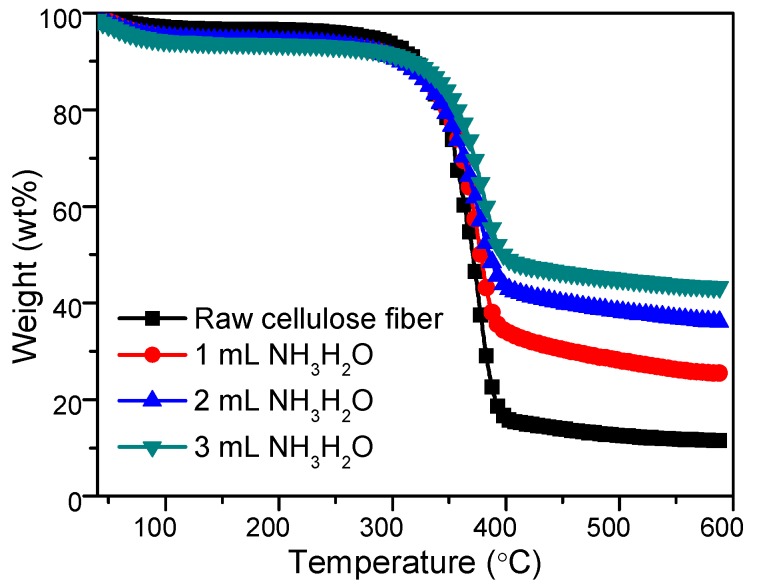
TGA curves of raw cellulose fibers and cellulose fibers catalyzed with different amounts of ammonia.

TGA curves was used to determine the amount of SiO_2_ modified on cellulose fibrex, shown in Figure 5. The residue of neat cellulose fibers was 11.33 wt.%, which was due to the inorganic ash content [42]. For the cellulose fibers after in situ hydrolysis of TEOS, the residue increased to 25.43 wt.%, 36.09 wt.% and 44.02 wt.%, with the amount of ammonia increasing from 1 mL to 2 mL and 3 mL, indicating that silica was successfully grafted to the surface of fibers. Besides, combined with SEM analyses, we can see that, adjusting the amount of NH_3_•H_2_O could well control the surface roughness of cellulose fibers and the silica content on the fiber surface.

### 3.2. Surface Wettability Property

In order to prove that the multi-scale hierarchical roughness structure on cellulose fiber surface was beneficial to the hydrophobic property, the contact angles of different paper surface were investigated, shown in the inset of Figure 4. As is well-known, cellulose fibers-based papers are inherently hydrophilic; water can be easily wetted. After hydrophobically modified with a thin layer of HDTMS, the paper showed a water contact angle of 122.1° ± 1°. For the paper based on cellulose fibers@SiO_2_ catalyzed with 1 mL ammonia, the water contact angle increased to 134.1° ± 1°. With the increase of the surface roughness, such as the fibers@SiO_2_ catalyzed with 2 mL, the water contact angle changed to 151.3° ± 1° and 145.6° ± 2°, respectively. Superhydrophobic surfaces, with a water contact angle of 151.3° ± 1°, can be formed by simply adjusting the ammonia to 2 mL to achieve a multi-scale hierarchical structure.

**Figure 6:**
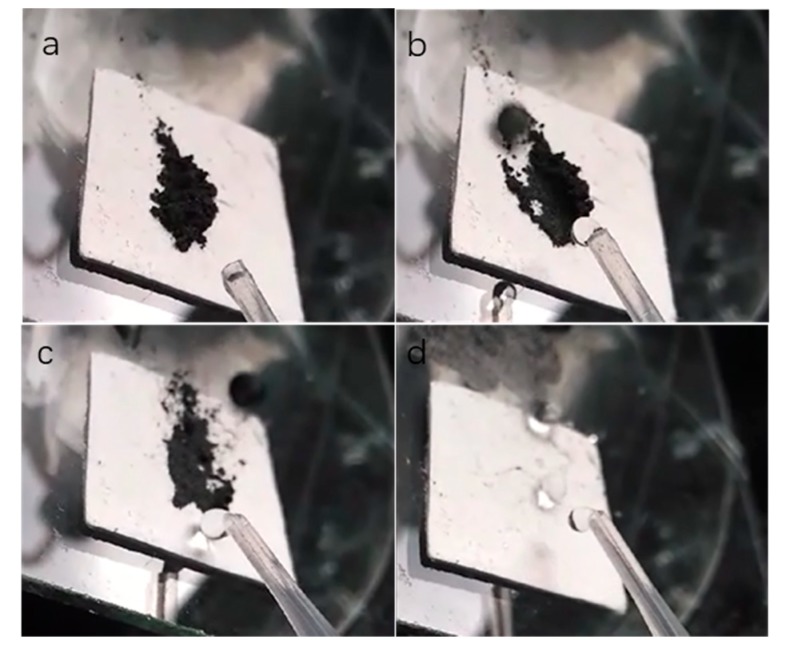
Sequential snapshots of self-cleaning performance of the as-prepared superhydrophobic paper (**a**–**d**).

**Figure 7:**
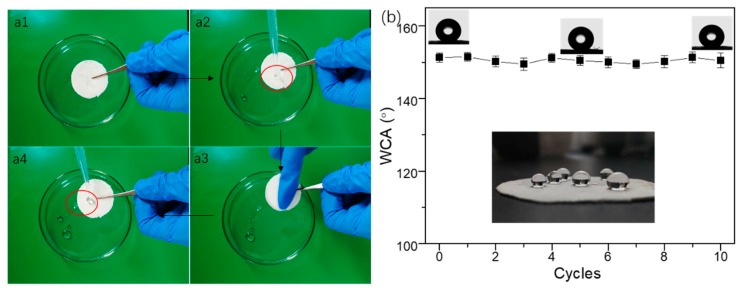
(**a1**–**a4**) Illustration of mechanical stability of the superhydrophobic coating; (**b**) The variation of water contact angles (CAs) of the as-prepared paper versus the number of finger-wipe cycles.

The as-prepared paper also had good self-cleaning properties from dirt removal tests, shown in Figure 6 and Video S1. As shown in Figure 6, a dirt removal test was carried out when an artificial dust (carbon powders) was put on the as-prepared paper, in order to observe self-cleaning properties of the coating. When cleaned by pouring water, the droplet took the dirt (carbon powder) away, leaving a thoroughly dry and clean surface, shown in Figure 6a–d and Video S1. Figure 7a and Video S2 displayed the method of finger abrasion test for the as-prepared superhydrophobic paper. The surface remained superhydrophobicity with a water contact angle of 150.8° even after 10 cycles finger abrasion (Figure 7b). The test indicates that the paper surface gained the nonwetting and self-cleaning properties after modification with SiO_2_ and subsequent HDTMS treatment.

## 4. Conclusions

A facile and environmentally friendly strategy for preparing biomimetic cellulose fibres@SiO_2_ composites was conducted by adding in-situ hydrolysis TEOS to cellulose pulps without any chemical treatment. Superhydrophobic paper could be further fabricated by using cellulose fibers@SiO_2_ as building blocks, followed by paper-making and the hydrophobization process. The as-prepared paper showed excellent superhydrophobic properties, with a water contact angle of 151.3° ± 1° and excellent self-cleaning properties against dirty contaminants. The preparation process is simple and controllable, and the product has excellent application foreground.

## Figures and Tables

**Table 1:** XPS elements contents of pristine Fibers and Fibers@SiO_2_ composites.

Entry	XPS (Atomic %)		
	C1s	O1s	Si2p
pristine Fibres	55.09	44.91	0
Fibres@SiO_2_	33.36	38.70	27.93

## Supplementary Materials

The following are available online at https://www.mdpi.com/1996-1944/12/9/1393/s1, Video S1: The self-cleaning performance of the as-prepared superhydrophobic paper. Video S2: The finger-wipe test to qualitatively evaluate the mechanical durability of the superhydrophobic paper.
